# Magnetic resonance imaging of pilonidal sinus disease: interobserver agreement and practical MRI reporting tips

**DOI:** 10.1007/s00330-023-10018-2

**Published:** 2023-08-11

**Authors:** Mohamed A. Abdelatty, Noha Elmansy, Mahmoud M. Saleh, Amany Salem, Sara Ahmed, Amr A. Gadalla, Mohamed F. Osman, Shady Mohamed

**Affiliations:** 1 Department of Diagnostic and Interventional Radiology, Kasr Al-Ainy Hospital, Cairo University Kasr Al-Ainy Street, Cairo, 11956 Egypt; 2 Department of Diagnostic and Interventional Radiology, National Cancer Institute, Cairo University, Cairo, Egypt; 3 Department of Public Health, Faculty of Medicine, Cairo University Kasr Al-Ainy Street, Cairo, 11956 Egypt; 4 Department of Radiation Oncology, University of Texas MD Anderson Cancer Center, 1515 Holcombe Blvd, Houston, TX 77030 USA

**Keywords:** Pilonidal sinus, Magnetic resonance imaging, Reliability

## Abstract

**Objective:**

To evaluate the interobserver agreement for the features of natal cleft pilonidal sinus disease (PSD) on magnetic resonance imaging (MRI) and propose a standardized checklist for reporting PSD on MRI.

**Materials and methods:**

Forty MRI studies of 39 discrete patients with PSD were retrospectively evaluated by five independent radiologists using a standardized checklist. Fleiss’ Kappa (*k*) coefficients of agreement were used to test the agreement between categorical variables. The MRI features of the natal cleft sepsis associated with PSD were classified into four main categories: morphology, branching and extensions, external skin openings, and the relationship of the PSD to the coccyx. A survey was created and disseminated online among general surgeons who treat patients with PSD to assess the relevance of the MRI features proposed in the standardized checklist.

**Results:**

The overall agreement regarding the identification of morphology of the natal cleft sepsis was moderate (*k* = 0.59). Lateral and caudal extensions interobserver agreement was substantial (*k* = 0.64 and 0.71, respectively). However, the overall agreement regarding the individual parts of anal sphincter involved was moderate (*k* = 0.47). Substantial interobserver agreement was found in assessing the proximity of the PSD to the coccyx (*k* = 0.62).

**Conclusion:**

Preoperative MRI can delineate the extensions and branching of PSD with substantial agreement. MRI is superior in describing the deep extensions of PSD with better reliability than assessing the number and locations of the external openings. Expert consensus agreement is needed to establish the MRI features necessary for optimal reporting of PSD.

**Clinical relevance statement:**

MRI can offer valuable information about the extent of sepsis associated with pilonidal sinus disease, particularly in cases with involvement of critical anatomical structures such as the coccyx and anal triangle. MRI can potentially contribute to more accurate patient stratification and surgical planning.

**Key Points:**

*• The interobserver agreement for assessing PSD’s lateral and caudal extension on MRI is substantial.*

*• MRI can describe deep extensions and branching of PSD with superior reliability than assessing the number and site of external openings.*

*• Reporting the relationship between natal cleft sepsis in PSD and the anal region may influence the surgical approach and postoperative healing.*

**Supplementary information:**

The online version contains supplementary material available at 10.1007/s00330-023-10018-2.

## Introduction

Pilonidal sinus disease (PSD) is a recurrent suppurative condition that commonly affects the natal cleft region in young patients of both sexes [1]. Its prevalence in the general population is approximately 26 out of 100,000 [2], with a higher incidence among males, hirsute, and obese individuals [3]. While PSD was previously believed to be congenital, the current prevailing theory suggests that loose hairs invade the skin surface, triggering a foreign body reaction that leads to inflammation and infection within the hair follicle, resulting in the characteristic midline pit necessary for diagnosis [4].

Different terms such as pilonidal cyst, abscess, or sinus are used interchangeably to describe this condition. PSD serves as an umbrella term covering various abnormalities and stages of presentation, including acute pilonidal abscess, chronic pilonidal sinus, and recurrent or complex pilonidal sinus. However, there is a lack of consensus on a classification system for the appearance of PSD [5, 6]. A recent systematic review of surgical classification systems for PSD noted that the location and number of sinuses defined those classifications but lacked evidence to support their use [6].

PSD is clinically diagnosed and typically requires surgical intervention. Historically, excision and healing by primary closure or secondary intention (“left-open”) have been common surgical approaches. Several other surgical options are available, including less invasive techniques and phenol injection [7]. The management of complications, particularly recurrences, poses a significant challenge for surgeons and can result from inadequate excision, delayed wound healing, or wound infection [7]. Preoperative mapping of PSD extensions is crucial for controlling natal cleft sepsis and ensuring proper drainage and excision of the sinus [8]. Intraoperative mapping using methylene blue is frequently performed. However, a study suggested that its use for guiding PSD surgery may lead to inadequate excision without evidence of reducing recurrence rates [9].

Imaging plays a limited role in PSD. Few studies have evaluated the use of ultrasonography (US) to assess the extensions of PSD for better excision and reduced recurrence rates. Superficial US has been suggested as an accurate modality for mapping the sepsis but proved more beneficial in distinguishing PSD from perianal fistulas [10, 11]. A recent consensus statement recommended MRI as a valuable tool for investigating complex PSD cases, especially in differentiating it from fistula in-ano [12]. Additionally, a study found MRI to be sensitive in distinguishing PSD and fistula-in-ano, suggesting that MRI can detect sepsis in both conditions with similar accuracy [13]. This study also found that, although perianal fistula and PSD share similar features on MRI, the absence of intersphincteric sepsis and internal opening in PSD distinguishes the two conditions. Preoperative MRI has been established as the standard of care for evaluating perianal fistulas based on several highly cited outcome studies [14–17], yet its use in PSD has not been widely adopted.

While there is extensive literature on PSD in terms of clinical data and surgical management of complicated recurrent cases, there is a notable lack of indexed medical literature on how to report PSD on MRI. Accordingly, we aimed to investigate interobserver agreement in identifying PSD on MRI and offer a practical guide for radiologists reporting PSD on MRI, highlighting the necessary information that surgeons require before surgery.

## Materials and methods

Institutional Review Board approval was obtained. Need for consent was waived due to the retrospective nature of the study and the fact that the data presented would be anonymized.

### Patients

Electronic records of all patients undergoing MRI fistulography for natal cleft sepsis at a tertiary referral hospital (*n* = 251) from January 2018 to December 2019 were reviewed using the local PACS system in the radiology department. Patients with a clinical diagnosis of pilonidal sinus disease were included, yielding 40 MRI studies of 39 discrete patients (36 male, 3 female). Patients with fistula-in-ano (*n* = 209) and hidradenitis suppurativa (*n* = 2) were excluded.

### MR imaging

All patients were imaged on one of two 1.5-T MRI machines (Achieva or Intera, Philips Medical System) in the supine position using a pelvic phased-array coil. No oral or intravenous contrast agent was administered. T2–weighted images turbo spin-echo sequences were acquired in three planes: axial, coronal, and sagittal (repetition time ms/echo time ms (TR/TE) 4000/100, field of view (FOV) 240–260 mm, slice thickness 2–4 mm, gap 0–0.5 mm, number of signals acquired 2, flip angle 90, matrix 512 × 512). STIR (short tau inversion recovery) weighted images were acquired in three planes: axial, coronal, and sagittal (repetition time ms/echo time ms (TR/TE) 2600/60, inversion time 150 ms, field of view (FOV) 240–260 mm, slice thickness 2–4 mm, gap 0–0.5 mm, number of signals acquired 2, flip angle 90, matrix 512 × 512).

### Image analysis

MRI studies were anonymized, and image analysis was done using RadiAnt DICOM viewer (Medixant). Five radiologists independently analyzed the images. One reader was a radiologist specialized in lower gastrointestinal (GI) and pelvic floor imaging (M.A.) with eight years of radiological experience. Two GI radiologists (N.M. and S.M.), each with nine years of radiological experience, and two general radiologists (S.A. and M.S.), each with eight years of radiological experience. The readers were blinded to the age, complaint, and clinical findings, but they were aware that all cases had natal cleft sepsis. Based on a recent systematic review of surgical classifications of PSD [6], a standardized checklist (Table 1) was formulated by a pelvic floor radiologist (M.A.) under the supervision of two senior GI radiologists (A.G. and M.O.) with 14 and 19 years of radiological experience, respectively. The checklist was transferred to an Excel spreadsheet (Microsoft Corporation). The data were represented as a drop-down list from which the readers could select the most appropriate choice to describe the natal cleft sepsis, as illustrated in Table 1. All measurements were standardized in millimeters. Prior to image analysis, an introductory meeting was held to clarify and discuss the checklist and define the different MRI features of PSD.

**Table 1:** Explanation of the checklist of the different features of the PSD on MRI

	**Yes**	**No**	**Additional data**
**Morphology** (select the **best** appropriate description of the natal cleft sepsis from the below choices, definitions are between the parentheses)			
Tract (tubular) (rough approximate length > 30 mm)			
Sinus cyst/cyst like (rounded/ovoid/short linear of high T2/STIR signal)			
Abscess (regular/irregular fluid collection) ± cellulitis			
**Skin openings** (identify the number of sinus skin openings, if more than one opening pick multiple, if multiple mention number, identify if all openings are within the navicular area)			
Single/multiple skin openings (mention number if multiple)			
All openings within navicular area			
**Extensions and branching** (mark yes/no at **each** feature to **best** describe of the extension and branching of the natal cleft sepsis from the below choices, definitions are between the parentheses)			
Is there **lateral** extension of sepsis (extension to gluteal region)?			
Is there **cranial** (subcutaneous) extension of the sepsis?			
Is there **caudal** extension of the sepsis (beyond the coccyx)?			
If caudal, is the sepsis reaching **anal sphincter complex ASCX?** If yes reaching (select only one): - External anal sphincter (EAS) - Internal anal sphincter (IAS) - Intersphincteric plane (ISP)			
**Proximity of sepsis to coccyx**			
Asses the relation of the sinus to coccyx/sacrum (distance less than or equal 10 mm = yes and mention distance in mm in additional data)			
Abnormal coccygeal/sacral signal of osteomyelitis (high STIR signal intensity)			

**Fig. 1:**
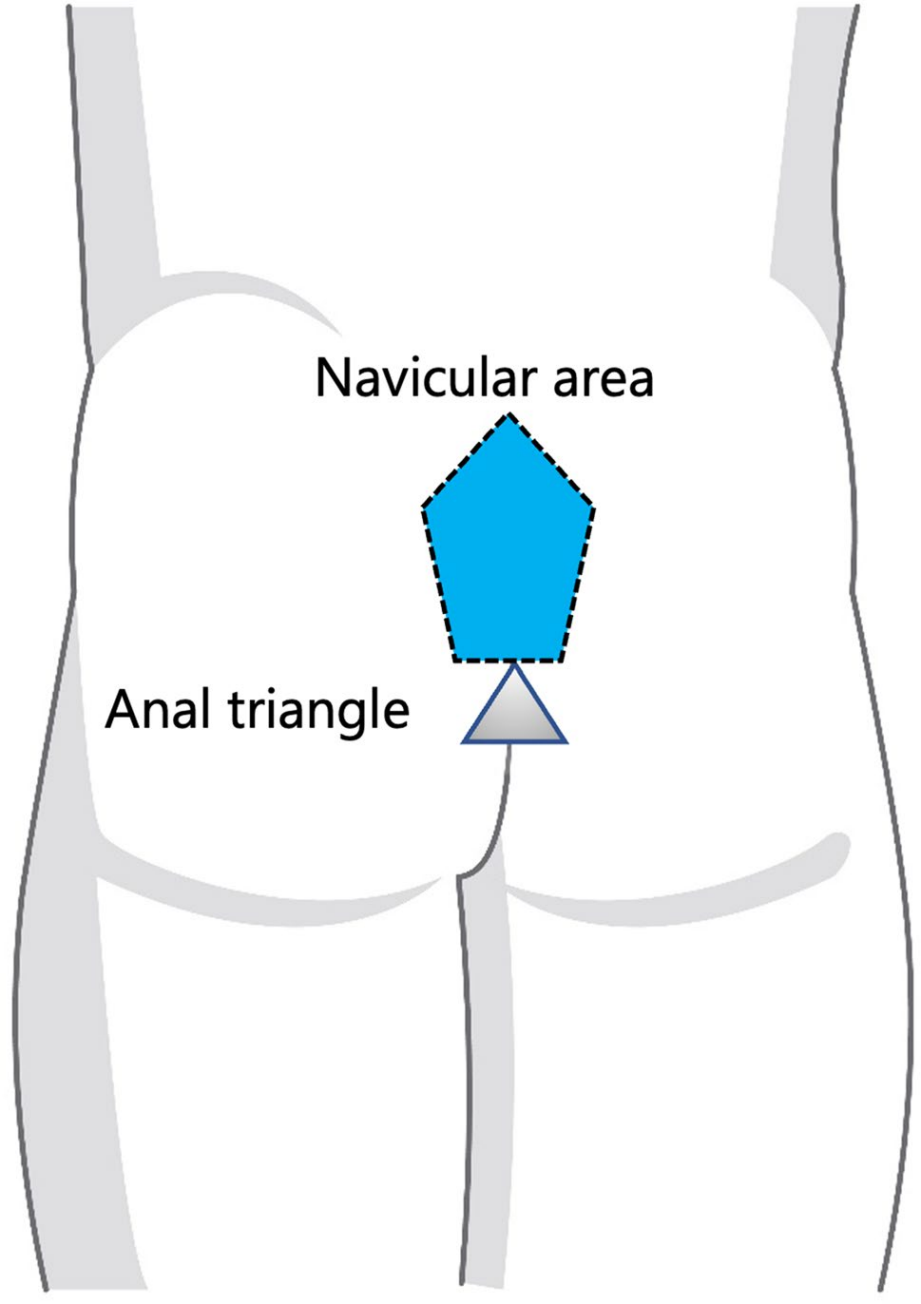
Surface anatomy of the buttocks showing the boundaries of the navicular area and the anal triangle

The natal cleft was identified as the midline intergluteal groove extending from the mid-sacral level blending inferiorly with the perineum [18]. The navicular area was defined as a ship-shaped region bounded laterally by the edges of the natal cleft and inferiorly by the apex of the anal triangle corresponding to the tip of the coccyx [19] (Fig. 1). Natal cleft sepsis indicative of PSD was recognized on MRI by the presence of high T2/STIR signal intensity at the midline or paramedian position within the natal cleft.

Morphological features of sepsis were described as either (i) “cyst/cyst-like” defined as a rounded/ovoid/short linear fluid signal, (ii) a “tract” defined as a longitudinal tubular structure roughly more than or equal 30 mm, or (iii) an “abscess” defined as an irregular fluid collection with or without surrounding cellulitis. The number of external skin openings was identified, with their location in relation to the navicular area noted as well.

Extension of the sepsis was described as “cranial” if extending cephalad within the subcutaneous tissue overlying the coccyx and/or sacrum. “Caudal” extension of the sepsis was defined if extending caudad beyond the tip of the coccyx. Additional note was made if the sepsis reached the anal sphincter complex (ASCX), whether it involved the external anal sphincter (EAS), extended beyond it to the intersphincteric plane (ISP), or reached the internal anal sphincter (IAS) [12]. “Lateral” extension referred to the extension of the sepsis beyond the lateral edges of the natal cleft reaching the gluteal region.

The proximity of sepsis to the coccyx was assessed if less than 10 mm. Note of abnormally elevated STIR signal intensity of the coccyx was suggestive of possible extension of sepsis deeply causing osteomyelitis [20].

### Clinical survey

To evaluate the clinical relevance of the MRI features proposed in the standardized checklist and determine if they could potentially impact surgical management, a survey was designed and disseminated online using Google Forms (Google.com). It was conducted among general surgeons who treat patients with PSD. Details of the survey form and the results obtained can be found in Supplementary Material 1.

### Statistical analysis

Data were coded and entered using the Statistical Package for the Social Sciences (SPSS) version 28 (IBM Corporation). Data were summarized using mean, standard deviation, median, minimum, and maximum for quantitative data and using frequency (count) and relative frequency (percentage) for categorical data. Fleiss’ Kappa measure of agreement was used to test agreement between categorical variables. The value of Kappa (*k*) was used as an indicator of the strength of agreement where the Kappa statistics were interpreted as follows: 0.01–0.20 (slight agreement), 0.21–0.40 (fair agreement), 0.41–0.60 (moderate agreement), 0.61–0.80 (substantial agreement), and 0.81–0.99 (almost perfect agreement) [21]. Testing for inter-rater reliability in numerical data was done using the intra-class coefficient (ICC) and Cronbach’s alpha reliability coefficient with their 95% confidence interval (95% CI). *p*-values less than 0.05 were considered statistically significant.

## Results

### Study population

Forty MRI studies of 39 discrete patients were included: 36 male and 3 female. The mean age at the time of MRI was 31 years (age range 16–57 years). One patient underwent a repeat examination after 6 months with an additional finding. Symptoms at the time of MRI examination were natal cleft discharge in all patients (100%). Twenty-five (64%) patients were imaged preoperatively, while 14 patients (36%) had recurrent natal cleft discharge following surgery for PSD. The agreements on the different features of natal cleft sepsis of the 40 MRI studies between the 5 raters are illustrated in Table 2. The count and percentage of each individual rater of the different features of natal cleft sepsis are summarized in Supplementary Material 2.

**Table 2:** Agreement on the different features of natal cleft sepsis of the 40 MRI studies between the 5 raters

	**Kappa (95% confidence interval)**	***p*-value**
**Morphology**		
* Overall agreement*	0.594 (0.515–0.673)	0.00
* Tract*	0.556 (0.458–0.654)	0.00
* Cyst*	0.596 (0.498–0.694)	0.00
* Abscess*	0.50 (0.596–0.792)	0.00
**Skin openings**		
* Overall agreement*	0.270 (0.192–0.347)	< 0.001
Single	0.247 (0.149–0.345)	< 0.001
Multiple	0.355 (0.257–0.453)	< 0.001
Cannot identify	0.139 (0.041–0.237)	0.005
* All openings within navicular area*	0.033 (–0.065 to 0.131)	0.513
**Extensions and branching**		
* Lateral extension*	0.643 (0.545–0.741)	0.00
* Cranial extension*	0.237 (0.139–0.335)	< 0.001
* Caudal extension*	0.712 (0.614–0.810)	0.00
* Reaching ASCX*	0.739 (0.641–0.837)	0.00
* If yes, reaching which part of ASCX (overall agreement)*	0.477 (0.411–0.543)	< 0.001
o Reaching EAS	0.218 (0.120–0.316)	< 0.001
o Reaching IAS	0.423 (0.325–0.521)	0.00
o Reaching ISP	0.069 (–0.029 to 0.167)	0.167
**Proximity to coccyx**		
* Overall agreement*	0.626 (0.528–0.724)	0.00
* Abnormal coccygeal signal intensity*	0.673 (0.575–0.771)	0.00

*p-*values less than 0.05 were considered as statistically significant

*ASCX* anal sphincter complex, *EAS* external anal sphincter, *IAS* internal anal sphincter, *ISP* intersphincteric plane

### Agreement on morphology

The overall agreement among the readers regarding the identification of morphology of the natal cleft sepsis was moderate (*k* = 0.59, 95% CI = 0.51–0.67). Similarly, the agreements for each independent category of morphology, i.e., tract, cyst, and abscess, were also moderate with kappa values of 0.55, 0.59, and 0.50, respectively.

### Skin openings

For the number of sinus skin openings depicted on MRI, the overall agreement between the different readers was found to be fair with kappa of 0.27 (95% CI = 0.19–0.34). Similarly, the agreements on single versus multiple openings were fair with *k* = 0.24 and 0.35, respectively. The identification of the skin openings within the navicular area yielded slight agreement with a kappa value of 0.03.

### Branching and extensions

There was substantial agreement among the different readers regarding the caudal extension of sepsis beyond the coccygeal tip with kappa of 0.71 (95% CI = 0.61–0.81). Similarly, the agreement of extension of the sepsis to reach the ASCX was also substantial, with a slightly higher kappa of 0.73. However, the overall agreement regarding the identification of the part of the sphincter involved was moderate (*k* = 0.47), with fair agreement for the involvement of the EAS (*k* = 0.21), moderate agreement for the involvement of the IAS (*k* = 0.42), and slight agreement for the ISP (*k* = 0.06). Further analysis of the inter-rater agreement of the caudal extension and the involvement of the ASCX based on the subspecialties of the readers is available in Supplementary Material 2*.*

The overall agreement for the lateral extension of the natal cleft sepsis to the gluteal region was substantial, with kappa of 0.64. However, the agreement for cranial (subcutaneous) extension between the readers was fair (*k* = 0.23).

### Relationship to the coccyx

The overall agreement regarding the proximity of natal cleft sepsis to the coccyx was substantial (*k* = 0.62). Similarly, there was substantial agreement in the identification of the coccygeal abnormal signal suggestive of osteomyelitis (*k* = 0.67). However, for the measurements of the distance of PSD from the coccyx, the ICC was 0.22 (95% CI = 0.01–0.56), *p*-value = 0.018, and Cronbach’s alpha = 0.59 (95% CI = 0.059–0.87), *p*-value = 0.018. Additional details of the measurements of the PSD relationship to the coccyx are available in Supplementary Material 2.

### Clinical survey

A summary of the answers recorded from the 48 surgeons who participated in the online survey is illustrated in Table 3.

**Table 3:** Summary of the survey showing count and percentage of surgeons and their answers. Total number of surgeons who participated were 48 surgeons who attend patients with PSD

*Would you be interested to know:*	Yes, it could change the surgical decision	Yes, but it won’t necessarily alter the surgical decision	No, it wouldn’t matter
The morphology of the pilonidal sinus; whether it is cystic, tubular, abscess?	15 (31.3%)	17 (35.4%)	16 (33.3%)
If there is cranial extension of the PSD along the subcutaneous plane?	19 (39.6%)	18 (37.5%)	11 (22.9%)
If there is caudal extension of the pilonidal sinus to the anal triangle and defining the extent of the sepsis in relation to the anal sphincter complex?	41 (85.4%)	4 (8.3%)	3 (6.3%)
If there is lateral extension of the pilonidal sinus to the gluteal region?	27 (56.3%)	12 (25.0%)	9 (18.8%)
The number of external skin openings of the pilonidal sinus and site of openings?	9 (18.8%)	19 (39.6%)	20 (41.7%)
The proximity of the pilonidal sinus to the sacrum/coccyx?	12 (25.0%)	19 (39.6%)	17 (35.4%)
If there are changes in the MRI signal intensity of the adjacent sacral/coccygeal parts suggestive of osteomyelitis?	25 (52.1%)	7 (14.6%)	16 (33.3%)

## Discussion

The results of our study revealed that the overall agreement for the extensions, branching, and relationship to the coccyx of the PSD using MRI was substantial. However, the overall agreement regarding the morphology of sepsis was moderate, and the agreement on the number and site of skin openings was fair to slight.

**Fig. 2:**
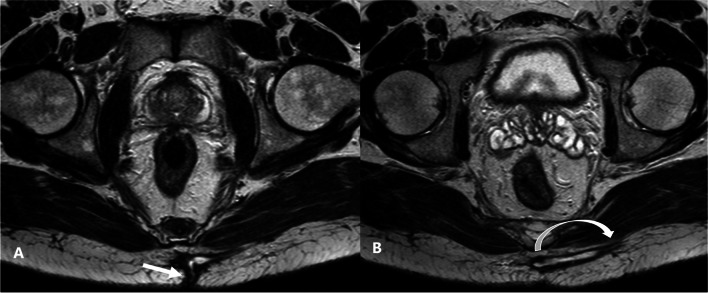
Axial T2-weighted image of the natal cleft of a 43-year-old male patient complaining of recurrent natal cleft discharge: (**A**) midline skin opening (arrow), (**B**) left natal cleft lateral branching to the subcutaneous gluteal region (curved arrow)

PSD originates subcutaneously and can extend along the fat planes cranially in the retro-coccygeal region, laterally to the gluteal region, and even caudally to reach the anal triangle [13]. We found substantial interobserver reliability regarding the presence of lateral extensions. A lateral extension of sepsis usually indicates a more complex process and could potentially alter the surgical plan to include a “rhomboid” excision with additional flap, especially if bilateral, to ensure adequate drainage and shorten healing time [22]. Consequently, we believe that radiological reports of PSD should include a comment on lateral extension/branching and its length from the natal cleft (Fig. 2).

**Fig. 3:**
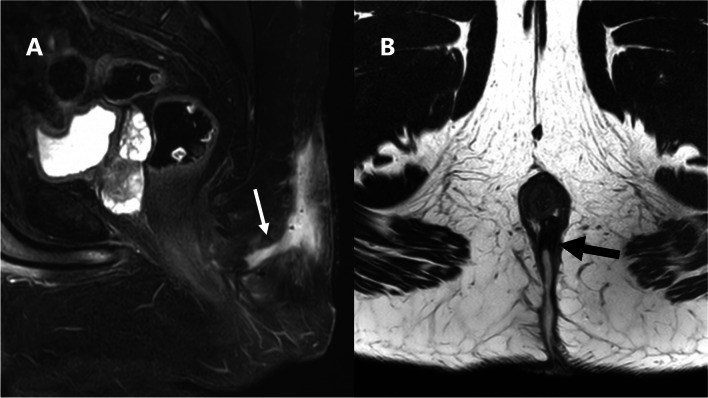
Sagittal STIR-weighted image (**A**) and axial T2-weighted image (**B**) of a 25-year-old patient complaining of recurrent natal cleft discharge after repeated operative interventions. **A** showing a large retro-coccygeal fluid collection with a caudal branch extending anteriorly within the posterior aspect of the perianal region, reaching the sphincteric complex (white arrow). **B** shows the caudal branch traversing the midline aspect of the deep external sphincter opposite 6 o’clock position to reach the left posterior quadrant of the intersphincteric plane, yet no definite penetration of the internal anal sphincter (black arrow)

When sepsis extends beyond the caudal tip of the coccyx and contacts the ASCX, confusion may arise regarding its origin, potentially overlapping with the diagnosis of perianal fistula [13]. Furthermore, a recent study listed the distance from the upper and lower edges of the PSD and the upper edge of the EAS on MRI as a possible prognostic factor after flap surgical techniques [23]. We found that the overall interobserver agreement regarding the caudal extension beyond the coccyx and the extension to reach the ASCX was substantial. However, the individual agreement for the different parts of the ASCX involved was moderate to slight, likely due to the different experiences of the raters and their familiarity with the ASCX anatomy. Additionally, 85% (41/48) of the surgeons who participated in our survey agreed that knowing the extension of the PSD to the anal triangle and its relationship to the ASCX could alter the surgical decision. This is consistent with a previously published survey of surgeons where the majority concurred that the proximity of the sepsis to the anal canal, although clinically challenging to determine, affects wound healing and impacts the choice of treatment [24]. Hence, we suggest that MRI reporting of PSD should include a comment on the caudal extension of the PSD beyond the coccyx, mention its relation to the anal triangle, and specify the individual parts of the ASCX involved if present (Fig. 3).

Conversely, the interobserver agreement for the identification of the cranial extension was only fair (*k* = 0.23), likely due to the absence of an anatomical landmark to define where the sepsis extends cranially. Therefore, we suggest the inclusion of the upper border of the PSD using the opposing sacral/coccygeal parts as a reference, in addition to the length of the sepsis.

**Fig. 4:**
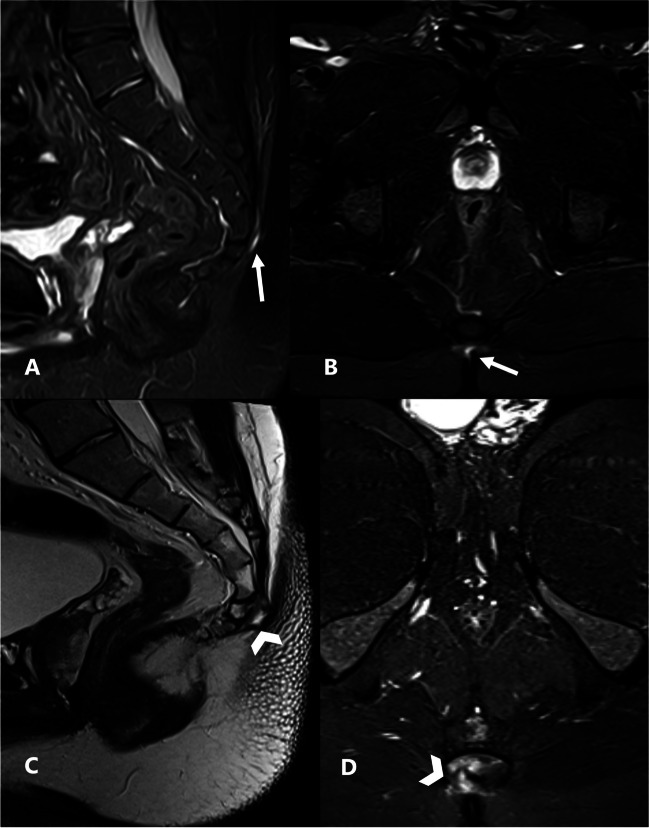
Pilonidal sinus disease in a 27-year-old male patient who underwent two consecutive MRI examinations 6 months apart with no interval surgical intervention. **A** and **B**: Sagittal and axial STIR-weighted images, respectively, of the first MRI showing a fine tract of fluid signal intensity (white arrows) seen near the posterior surface of the first coccygeal segment with normal signal of the coccyx. **C** and **D**: Sagittal T2- and axial STIR-weighted images, respectively, from the MRI performed 6 months later showing progression in size to form a small collection associated with newly developed abnormally elevated STIR signal of the adjacent coccygeal segment (arrowhead) suggestive of developing osteomyelitis of the coccyx

In terms of changes in coccygeal signal and proximity of sepsis to the coccyx, the interobserver agreement was substantial. Although coccygeal osteomyelitis secondary to PSD is a relatively rare complication, we believe that radiologists should be mindful of this serious complication and report it if present. Such findings may lead to a change in surgical decision, potentially requiring drastic procedures like coccygectomy [25]. One of the patients included in our study underwent two MRI scans with a 6-month interval between the two studies, following antibiotic treatment without surgical intervention. The second MRI revealed the development of an abnormal intrinsic signal in the coccyx, suggesting deep extension of the sepsis (Fig. 4).

**Fig. 5:**
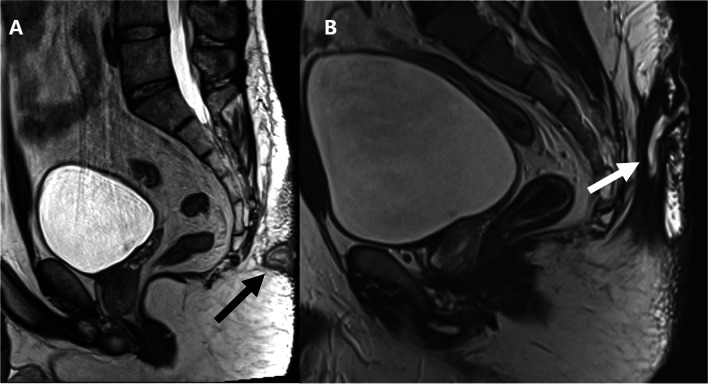
Sagittal T2-weighted images of two different patients with PSD; (**A**) a 22-year-old male with PSD showing rounded/cystic morphology; (**B**) a 30-year-old male with tubular branching PSD tract

**Fig. 6:**
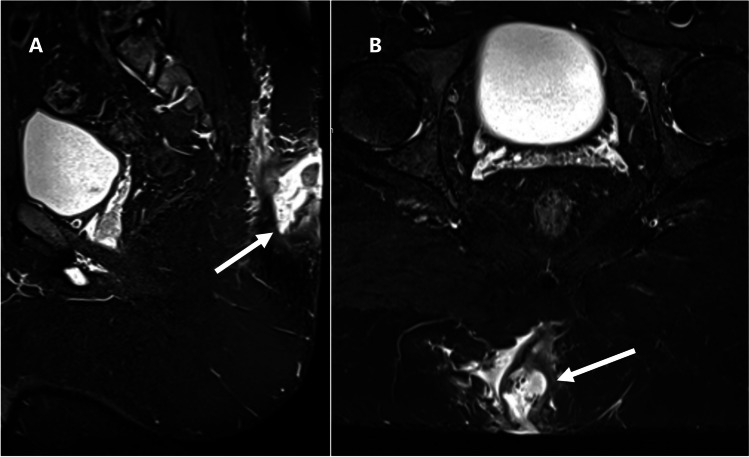
(**A**) Sagittal STIR-weighted image showing an irregular branching subcutaneous retro-coccygeal fluid collection with surrounding extensive inflammatory changes suggestive of cellulitis and presumed two skin openings. (**B**) Axial T2 WI image showing the collection posterior to the coccyx with no evidence of deeper extension

The overall agreement regarding the morphological appearance of PSD was only moderate. While for radiologists semantics are generally fundamental, for surgeons treating patients with PSD, apart from the transverse and longitudinal measurements, the morphological distinction between cyst and tract might not be of importance (Fig. 5). In our online survey, most of the surgeons (68%) replied that the morphology of PSD would likely not impact the surgical decision. However, the implication of an intergluteal abscess is crucial because that degree of inflammation can affect the surgical timing, impact healing, and possibly lead to recurrence of the sepsis [26]. Accordingly, we suggest that MRI reports of PSD should confirm or negate the presence of features of acute abscess and surrounding cellulitis (Fig. 6).

The overall interobserver agreement regarding the number of external skin openings was only fair, indicating limitations in identifying the skin openings of PSD on MRI. However, this shortcoming in imaging is unlikely to be significant since clinical examination can easily depict the skin openings. In fact, 81% of the surgeons in our survey believed that clinical examination would be sufficient or even superior in detecting the external openings of PSD. Similarly, the overall agreement regarding the presence of skin openings within the navicular area was slight, suggesting that the assessment of the navicular area cannot be simply adopted from surgical and clinical literature to MRI. Additional anatomical landmarks could aid in mapping the disease more accurately.

### Limitations

Our study does have limitations. As a retrospective study with no available surgical records of the included patients, we could not evaluate the possible alteration in the surgical planning or the effect on recurrences. Future studies are recommended to assess the impact of these MRI parameters on the surgical outcome and recurrences. Additionally, only one reader was specialized in pelvic floor imaging. While we believe this ensures the generalizability of our results, familiarity with ASCX anatomy could have improved agreement on the degree of involvement of the anal sphincter.

## Conclusion

To our knowledge, no prior studies have evaluated the inter-rater agreement for PSD on MRI or proposed a reporting checklist for this condition (Table 4). Preoperative MRI can delineate the extensions and branching of PSD with substantial agreement, particularly for caudal and lateral extensions, which indicate a more complex condition and may necessitate a modification in the surgical approach. MRI is superior in describing the deep extensions of sepsis with better reliability than assessing the number and locations of the external openings. Further validity studies correlating MRI findings with surgical outcomes and postoperative recurrences are recommended.

**Table 4:** Proposed structured reporting template for MRI of the pilonidal sinus disease

Structured reporting template for MRI of the pilonidal sinus disease
Dimensions Length _______ cm Width _______cm
Morphology Cystic Tubular Abscess _______ Associated MR features of cellulitis _______
Skin openings Single external opening _______ Multiple external openings _______ Number of identified external openings _______ All identified external openings within the navicular area _______
Branching and extensions Lateral extension _______ - Measurement from the midline _______ Cranial extension - Upper border at sacral/coccygeal level _______ Caudal extension - Inferior border at sacral/coccygeal level _______ Reaching anal sphincter complex _______ Part of the anal sphincter complex involved: - External anal sphincter - Intersphincteric plane - Internal anal sphincter
Relationship to coccyx PSD near the coccyx _______ Distance of PSD from the coccyx _______ Abnormal coccygeal signal intensity _______

Our study offers a foundation that can help create expert consensus agreement needed to establish the MRI features necessary for optimal MRI reporting of PSD. We believe that collaboration between radiologists and surgeons in developing a classification system for PSD can significantly enhance patient stratification, leading to tailored surgical management plans and reduced recurrence rates.

### Supplementary information

Below is the link to the electronic supplementary material.

Supplementary file1 (PDF 188 KB)

## Abbreviations

ASCX: Anal sphincter complex
CI: Confidence interval
EAS: External anal sphincter
IAS: Internal anal sphincter
ISP: Intersphincteric plane
MRI: Magnetic resonance imaging
PSD: Pilonidal sinus disease

## Declarations

### Guarantor

The scientific guarantor of this publication is Mohamed A. Abdelatty.

### Statistics and biometry

One of the authors (Amany Salem) has significant statistical expertise.

### Study subjects or cohorts overlap

Some of the cases were presented at the RSNA 2020 educational exhibit as an electronic poster titled “MR Imaging of Natal Cleft Sepsis: Pilonidal Sinus Disease and Mimics”.

### Methodology

- Retrospective
- Observational
- Performed at one institution
