# Supplementary material for: Magnetic resonance imaging of pilonidal sinus disease: interobserver agreement and practical MRI reporting tips
Source: Eur Radiol. 2023 Aug 11;34(1):115–25. doi: 10.1007/s00330-023-10018-2 (PMC10791724; doi:10.1007/s00330-023-10018-2)
Supplement: Supplementary file 1 — Supplementary file1 (PDF 188 KB) [file 330_2023_10018_MOESM1_ESM.pdf]

1. **Do you usually see patients with pilonidal sinus disease in your practice?**

| | | Frequency | Percent | Valid Percent | Cumulative Percent |
|---|---|---|---|---|---|
| Valid | Yes | 48 | 100.0 | 100.0 | 100.0 |

2. **When would you request MRI for a patient with Pilonidal Sinus Disease (PSD)?**

| | | Frequency | Percent | Valid Percent | Cumulative Percent |
|---|---|---|---|---|---|
| Valid | I refer all patients with PSD for MRI | 2 | 4.2 | 4.2 | 4.2 |
| | No, it's an unnecessary cost | 10 | 20.8 | 20.8 | 25.0 |
| | Only if there is clinical suspicion that the sepsis reached perianal region | 12 | 25.0 | 25.0 | 50.0 |
| | Only to exclude other causes of sepsis as perianal fistula or hidradenitis suppurativa | 9 | 18.8 | 18.8 | 68.8 |
| | Only when clinical examination is difficult | 5 | 10.4 | 10.4 | 79.2 |
| | Only when it's a recurrent/chronic patient | 10 | 20.8 | 20.8 | 100.0 |
| | Total | 48 | 100.0 | 100.0 | |

3. **Would you be interested to know the morphology of the pilonidal sinus; whether it is cystic, tubular or abscess?**

| | | Frequency | Percent | Valid Percent | Cumulative Percent |
|---|---|---|---|---|---|
| Valid | No, it would not matter | 16 | 33.3 | 33.3 | 33.3 |
| | Yes, but it won't necessarily alter the surgical decision | 17 | 35.4 | 35.4 | 68.8 |
| | Yes, it could possibly change the surgical decision | 15 | 31.3 | 31.3 | 100.0 |
| | Total | 48 | 100.0 | 100.0 | |

4. **Would you be interested to know if there is cranial extension of the pilonidal sinus along the subcutaneous plane?**

| | | Frequency | Percent | Valid Percent | Cumulative Percent |
|---|---|---|---|---|---|
| Valid | No, it would not matter | 11 | 22.9 | 22.9 | 22.9 |
| | Yes, but it won't necessarily alter the surgical decision | 18 | 37.5 | 37.5 | 60.4 |
| | Yes, it could change the surgical decision | 19 | 39.6 | 39.6 | 100.0 |
| | Total | 48 | 100.0 | 100.0 | |

**5. Would you be interested to know if there is caudal extension of the pilonidal sinus to the anal triangle and defining the extent of the sepsis in relation to the anal sphincter complex?**

| | | Frequency | Percent | Valid Percent | Cumulative Percent |
|---|---|---|---|---|---|
| Valid | No, it would not matter | 3 | 6.3 | 6.3 | 6.3 |
| | Yes, but it won't necessarily alter the surgical decision | 4 | 8.3 | 8.3 | 14.6 |
| | Yes, it could change the surgical decision | 41 | 85.4 | 85.4 | 100.0 |
| | Total | 48 | 100.0 | 100.0 | |

**6. Would you be interested to know if there is lateral extension of the pilonidal sinus to the gluteal region?**

| | | Frequency | Percent | Valid Percent | Cumulative Percent |
|---|---|---|---|---|---|
| Valid | No, it would not matter | 9 | 18.8 | 18.8 | 18.8 |
| | Yes, but it won't necessarily alter the surgical decision | 12 | 25.0 | 25.0 | 43.8 |
| | Yes, it could change the surgical decision | 27 | 56.3 | 56.3 | 100.0 |
| | Total | 48 | 100.0 | 100.0 | |

**7. Would you be interested to know the number of external skin openings of the pilonidal sinus?**

| | | Frequency | Percent | Valid Percent | Cumulative Percent |
|---|---|---|---|---|---|
| Valid | Maybe but clinical examination is likely to be superior | 20 | 41.7 | 41.7 | 41.7 |
| | No, clinical examination is sufficient | 19 | 39.6 | 39.6 | 81.3 |
| | Yes | 9 | 18.8 | 18.8 | 100.0 |
| | Total | 48 | 100.0 | 100.0 | |

**8. Would you be interested to know the proximity of the pilonidal sinus to the sacrum/coccyx?**

| | | Frequency | Percent | Valid Percent | Cumulative Percent |
|---|---|---|---|---|---|
| Valid | No, it wouldn't matter | 17 | 35.4 | 35.4 | 35.4 |
| | Yes, but it won't necessarily alter the surgical decision | 19 | 39.6 | 39.6 | 75.0 |
| | Yes, it could change the surgical decision | 12 | 25.0 | 25.0 | 100.0 |
| | Total | 48 | 100.0 | 100.0 | |

**9. Would you be interested to know if there are changes in the MRI signal intensity of the adjacent sacral/coccygeal parts suggestive of osteomyelitis?**

| | | Frequency | Percent | Valid Percent | Cumulative Percent |
|---|---|---|---|---|---|
| Valid | No, it wouldn't matter | 16 | 33.3 | 33.3 | 33.3 |
| | Yes, but it won't necessarily alter the surgical decision | 7 | 14.6 | 14.6 | 47.9 |
| | Yes, it could change the surgical decision | 25 | 52.1 | 52.1 | 100.0 |
| | Total | 48 | 100.0 | 100.0 | |

## 1. Count and percentage of each individual rater for the different features of the natal cleft sepsis

| | | Rater 1 | | Rater 2 | | Rater 3 | | Rater 4 | | Rater 5 | |
|---|---|---|---|---|---|---|---|---|---|---|---|
| | | **Count** | **%** | **Count** | **%** | **Count** | **%** | **Count** | **%** | **Count** | **%** |
| **Morphology** | **Tract** | 21 | 52.5% | 16 | 40.0% | 22 | 55.0% | 18 | 45.0% | 13 | 32.5% |
| | **Cyst** | 16 | 40.0% | 19 | 47.5% | 13 | 32.5% | 19 | 47.5% | 23 | 57.5% |
| | **Abscess** | 3 | 7.5% | 5 | 12.5% | 5 | 12.5% | 3 | 7.5% | 4 | 10.0% |
| **Skin openings** | **Single** | 24 | 60% | 24 | 60.0% | 21 | 52.5% | 24 | 60.0% | 31 | 77.5% |
| | **Multiple** | 13 | 32.5% | 15 | 37.5% | 10 | 25.0% | 14 | 35.0% | 4 | 10.0% |
| | **Can't identify** | 3 | 7.5% | 1 | 2.5% | 9 | 22.5% | 2 | 5.0% | 5 | 12.5% |
| **Number of skin opening if multiple** | **NA** | 27 | 67.5% | 25 | 62.5% | 30 | 75.0% | 26 | 65.0% | 36 | 90.0% |
| | **2** | 7 | 17.5% | 6 | 15.0% | 5 | 12.5% | 8 | 20.0% | 3 | 7.5% |
| | **3** | 5 | 12.5% | 7 | 17.5% | 4 | 10.0% | 4 | 10.0% | 1 | 2.5% |
| | **4** | 1 | 2.5% | 2 | 5.0% | 1 | 2.5% | 2 | 5.0% | 0 | 0.0% |
| **All skin openings within navicular area** | **Yes** | 37 | 92.5% | 36 | 90.0% | 37 | 92.5% | 30 | 75.0% | 28 | 70.0% |
| | **No** | 3 | 7.5% | 4 | 10.0% | 3 | 7.5% | 10 | 25.0% | 12 | 30.% |
| **Lateral extension** | **Yes** | 8 | 20.0% | 9 | 22.5% | 8 | 20.0% | 6 | 15.0% | 7 | 17.5% |
| | **No** | 32 | 80.0% | 31 | 77.5% | 32 | 80.0% | 34 | 85.% | 33 | 82.5% |
| **Cranial extension** | **Yes** | 24 | 60.0% | 17 | 42.5% | 14 | 35.0% | 18 | 45.0% | 33 | 82.5% |
| | **No** | 16 | 40.0% | 23 | 57.5% | 26 | 65.0% | 22 | 55.0% | 7 | 17.5% |
| **Caudal extension** | **Yes** | 12 | 30.0% | 10 | 25.0% | 17 | 42.5% | 15 | 37.5% | 15 | 37.5% |
| | **No** | 28 | 70.0% | 30 | 75.0% | 23 | 57.5% | 25 | 62.5% | 25 | 62.5% |
| **Reaching ASCX** | **Yes** | 7 | 17.5% | 8 | 20.0% | 10 | 25.0% | 10 | 25.0% | 6 | 15.0% |
| | **No** | 33 | 82.5% | 32 | 80.0% | 30 | 75.0% | 30 | 75.0% | 34 | 85.0% |
| | **ISP** | 2 | 50.0% | 4 | 10.0% | 3 | 7.5% | 1 | 2.5% | 2 | 5.0% |

| | | | | | | | | | | | |
|---|---|---|---|---|---|---|---|---|---|---|---|
| **If yes, reaching which part of the ASCX?** | **IAS** | 0 | 37.5% | 3 | 7.5% | 5 | 12.5% | 6 | 15.0% | 2 | 5.0% |
| | **EAS** | 5 | 12.5% | 1 | 2.5% | 2 | 5.0% | 3 | 7.5% | 2 | 5.0% |
| **Proximity to coccyx** | **Yes** | 13 | 32.5% | 23 | 57.5% | 23 | 57.5% | 29 | 72.5% | 22 | 55.0% |
| | **No** | 27 | 67.5% | 17 | 42.5% | 17 | 42.5% | 11 | 27.5% | 18 | 45.0% |
| **Abnormal coccyx signal intensity** | **Yes** | 4 | 10.0% | 8 | 20.0% | 6 | 15.0% | 10 | 25.0% | 5 | 12.5% |
| | **No** | 36 | 90.0% | 32 | 80.0% | 34 | 85.0% | 30 | 75.0% | 35 | 87.5% |

*ACSX = anal sphincter complex, ISP = intersphincteric plane, IAS = internal anal sphincter, EAS = external anal sphincter*

**2. Inter-rater agreement for the extension of PSD to the anal sphincter complex according to subspeciality of readers**

| | **Kappa (95% confidence interval)** | ***p*-value** |
|---|---|---|
| ***Pelvic floor radiologist and GIT radiologists*** | | |
| *Reaching ASCX* | 0.792 (0.613-0.971) | 0.00 |
| *If yes, reaching which part of ASCX (overall agreement)* | 0.320 (0.131-0.510) | <0.001 |
| ○ Reaching EAS | 0.082 (-0.231-0.396) | 0.607 |
| ○ Reaching IAS | 0.462 (0.148-0.776) | 0.04 |
| ○ Reaching ISP | -0.011 (-0.325-0.303) | 0.945 |
| ***Pelvic floor radiologist and general radiologists*** | | |
| *Reaching ASCX* | 0.747 (0.568-0.926) | <0.001 |
| *If yes, reaching which part of ASCX (overall agreement)* | 0.125 (-0.060-0.310) | 0.186 |
| ○ Reaching EAS | -0.011 (-0.325-0.303) | 0.945 |
| ○ Reaching IAS | 0.133 (-0.181-0.447) | 0.405 |
| ○ Reaching ISP | -0.219 (-0.533-0.095) | 0.172 |
| ***GIT radiologists and general radiologists*** | | |
| *Reaching ASCX* | 0.682 (0.555-0.808) | 0.00 |
| *If yes, reaching which part of ASCX (overall agreement)* | 0.154 (0.022-0.286) | 0.022 |
| ○ Reaching EAS | 0.061 (-0.161-0.283) | 0.589 |
| ○ Reaching IAS | 0.214 (-0.08-0.436) | 0.059 |
| ○ Reaching ISP | -0.083 (-0.305-0.139) | 0.462 |

*ACSX = anal sphincter complex, ISP = intersphincteric plane, IAS = internal anal sphincter, EAS = external anal sphincter. P-values less than 0.05 were considered as statistically significant.*

## 3. Measurements of the distance of PSD from coccyx in cases showing proximity to the coccyx of each rater

| Distance of sepsis from the coccyx (mm) | Mean | Standard Deviation | Median | Minimum | Maximum |
|---|---|---|---|---|---|
| **Rater1** | 4.27 | 2.20 | 4.00 | 0.00 | 8.00 |
| **Rater 2** | 3.09 | 2.79 | 2.00 | 0.00 | 10.00 |
| **Rater 3** | 3.13 | 3.03 | 3.00 | 0.00 | 9.00 |
| **Rater 4** | 3.97 | 2.92 | 3.00 | 0.00 | 10.00 |
| **Rater5** | 4.82 | 2.59 | 4.00 | 1.00 | 9.00 |
